# The paradoxical role of emotional intensity in the perception of vocal affect

**DOI:** 10.1038/s41598-021-88431-0

**Published:** 2021-05-06

**Authors:** N. Holz, P. Larrouy-Maestri, D. Poeppel

**Affiliations:** 1 Department of Neuroscience, Max Planck Institute for Empirical Aesthetics, Frankfurt/M, Germany; 2 Max Planck NYU Center for Language, Music, and Emotion, Frankfurt/M, Germany; 3 Department of Psychology, New York University, New York, NY USA

**Keywords:** Human behaviour, Emotion

## Abstract

Vocalizations including laughter, cries, moans, or screams constitute a potent source of information about the affective states of others. It is typically conjectured that the higher the intensity of the expressed emotion, the better the classification of affective information. However, attempts to map the relation between affective intensity and inferred meaning are controversial. Based on a newly developed stimulus database of carefully validated non-speech expressions ranging across the entire intensity spectrum from low to peak, we show that the intuition is false. Based on three experiments (*N* = 90), we demonstrate that intensity in fact has a paradoxical role. Participants were asked to rate and classify the authenticity, intensity and emotion, as well as valence and arousal of the wide range of vocalizations. Listeners are clearly able to infer expressed intensity and arousal; in contrast, and surprisingly, emotion category and valence have a perceptual sweet spot: moderate and strong emotions are clearly categorized, but peak emotions are maximally ambiguous. This finding, which converges with related observations from visual experiments, raises interesting theoretical challenges for the emotion communication literature.

## Introduction

Whether conveyed by the face, body, or voice, expressions of emotion are ubiquitous. The inferred meaning of the expressions is, generally speaking, substantially aligned with the affective content expressed, and it is intuitive to suggest that the stronger the expressed affective state the more clear-cut the inferred emotional meaning. Indeed, a body of research suggests that high-intensity emotion expressions are better ‘recognized’^1–5^. Importantly, both discrete-emotion and dimensional theories predict this pattern of results, although by different mechanisms: either maximized distance to other emotions by the increasing recruitment of diagnostic content (e.g., facial muscle action^6,7^) or through maximized distance in the affective space encompassed by the dimensions valence and arousal^8^ (note that alternative and higher-dimension models arrive at similar predictions, e.g., Plutchik’s circumplex but discrete emotion view^9^). In other words, the prevailing approaches conjecture less confusion or ambiguity for the classification of highly intense expressions than for intermediate ones, as the distinctiveness of emotion expressions is predicted to increase with increasing intensity.

This generalization has been challenged by the discovery of perceptual ambiguity for facial^10,11^ and vocal^12^ expressions of peak emotional intensity. In the latter study, vocalizations of extreme positive valence could not be disambiguated from extreme negative valence. Moreover, these authors demonstrated a trend opposite the predicted relation for peak intense positive situations: the reactions of real-life lottery winners were rated more negatively as hedonic intensity (in this case cued by the prize sum) increased. They argue that peak emotion expression is *inherently* ambiguous and reliant on contextual information^12–14^.

The research on the ambiguity of intense expressions is intriguing, but key issues lack sufficient evidence to refine our theoretical understanding. The studies on peak emotion elegantly contrast positive and negative affect. As such, *one* aspect of affective experience (i.e., valence) is hard to differentiate. Valence along with arousal are thought to constitute essential building blocks of core affect^15,16^. Hence, its compromised perceptual representation invites the speculation that peak intense vocalizations do not convey *any* affective meaning. But it is not known whether arousal, an equally fundamental property of affect, is similarly indistinctive. Moreover, the data raise the question whether individual emotions of the same or opposing valence can be differentiated, or if only peak positive affect is unidentifiable.

These considerations are important to understand the complex role of emotion intensity. From an analytic perspective, the two types of studies yielding the contradictory evidence are difficult to compare. The contrast differs between the groups of studies (emotion categories versus hedonic value, i.e., positive or negative). Additionally, it is unclear whether ambiguity is specific to *peak* emotion, or if affective expressions are generally more ambiguous than previously thought^12,13^. The one group of studies largely base their interpretation on results obtained with moderately intense emotion expressions; peak intensity emotional states were not examined. On the other hand, the data challenging this interpretation exclusively address peak emotional states. In summary, the data motivating these ideas are too sparse to adjudicate between the theoretical alternatives.

Various questions arise. First, what underlies the perceptual ambiguity, that is, which aspects of emotion lack a differentiable perceptual representation? Are valence, arousal, and emotion category equally affected—and is ambiguity a general property of emotion communication? Second, how does affective information vary as a function of emotion intensity, if not linearly, as previously assumed—and what are the resulting theoretical implications? We illuminate the seemingly contradictory findings and provide insight into the processes of nonverbal emotion expression.

Nonverbal vocalizations reflect variable degrees of spontaneity, cognitive control, social learning, and culture^17,18^. They are largely shaped by physiological effects on voice. Such effects, associated with sympathetic activation or arousal, can be perceived through characteristic changes in vocal cues^19,20^ and play a role especially in the communication of strong emotion^21,22^. Specifically, for nonverbal expressions arising from extreme situations, little voluntary regulation and socio-cultural dependency are expected^23,24^. Emotionally intense vocalizations—in negative as well as positive contexts—oftentimes encompass harsh sounding call types such as screams, roars, and cries^23,25–27^. On a functional account, the characteristic acoustic structure (i.e., nonlinearities and spectro-temporal modulations) seem ideal to capture listener attention^25–27^. Importantly, acoustic signatures are linked to high attention and salience as well as the perception of arousal across species and signal modalities^26,28–33^. Their biological relevance thus seems irrefutable.

Though valence and arousal are equally fundamental in emotion theoretical frameworks, it is implausible to assume that the human voice does not signal physical activation or arousal in the most extreme instances of emotion. In fact, from an ethological perspective, a perceptual representation of arousal as well as the specific intensity of the emotional state seem essential, even when overall valence and the specific type of emotion cannot be identified.

**Figure 1:**
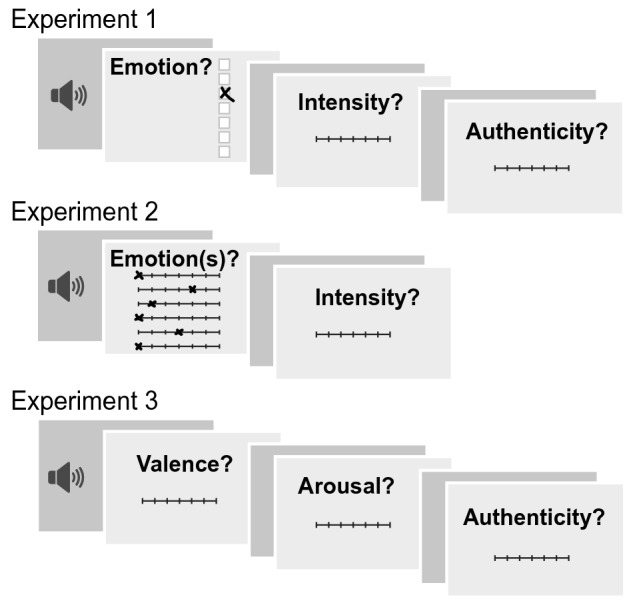
Experimental paradigm. Schematic of one experimental trial in each of the three tasks, the *emotion categorization task* (Experiment 1), the *emotion rating task* (Experiment 2), and the *dimensional rating task* (Experiment 3). The total session consisted of one practice block (4 trials) and ten experimental blocks (48 trials each), followed by a short questionnaire on sociodemographic information. All rating scales were end-to-end labeled 7-point Likert scales. The emotion labels were presented in random order across but fixed order within participants.

To address specifically the influence of emotional intensity on emotion perception, we use nonverbal vocalizations from a newly developed database, the *Variably Intense Vocalizations of Affect and Emotion Corpus* (VIVAE). The corpus, openly available (http://doi.org/10.5281/zenodo.4066235), encompasses a range of vocalizations and was carefully curated to comprise expressions of three positive (achievement/triumph, positive surprise, sexual pleasure) and three negative affective states (anger, fear, physical pain), ranging from low to peak emotion intensity. Perceptual evaluations were performed by *N* = 90 participants, who in three separate experiments classified (Experiment 1, Fig. 1) and rated emotion (Experiment 2—given the limitations of forced choice response formats, discussed, e.g., in Refs.^1,34^), rated the affective dimensions valence and arousal (Experiment 3), and rated perceived authenticity (Experiments 1 and 3). We hypothesized that listeners would be able to classify emotional categories significantly above chance (Experiments 1 and 2) and to rate the affective properties of the stimuli congruently with the expressed affective states (Experiment 3). The critical hypothesis was as follows: All judgments were examined as a function of emotion intensity, which we expected to have a systematic effect on stimulus classification (Experiment 1) and on perceptual ratings (Experiments 2 and 3). Following the theoretical frameworks, we predicted that intensity and arousal would be classified clearly over the range of expressed intensities, while, in line with recent empirical data, the amplifying role of emotional intensity on the classification of valence and emotion category would plateau at strong emotion. Peak emotion should be maximal in received intensity and arousal—however, valence and emotion category would be more ambiguous. Together, we conjectured a paradoxical effect of the intensity of expressed emotion on perception, a finding not easy to accommodate by current versions of categorical and dimensional theories of emotion.

## Results

### Emotions are accurately classified

**Figure 2:**
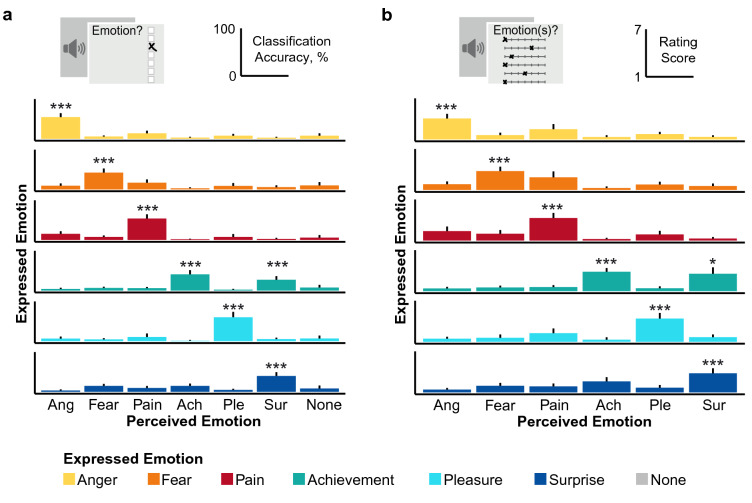
Emotion classification and rating patterns for each expressed emotion. (**a**) The main diagonal represents ‘correct’ emotion classification. The most common confusion is between achievement and surprise. Interestingly, this confusion is not perfectly symmetrical, as surprise, when misclassified, is as likely to be categorized as achievement or fear. (**b**) Average ratings on matching scales are higher than ratings on other scales. Scores on scales of matching valence are depicted in the upper left corner (negative) and the lower right corner (positive). Error bars indicate 95% confidence intervals. *Ach* Achievement, *Ang* anger, *Ple* pleasure, *Sur* surprise. *p < 0.05. ***p < 0.001. Figure created with R version 4.0.3^35^.

In the *emotion categorization task* (Expt1, Figs. 1 and 2a, Supplementary Table S2), classification was significantly better than chance (16.67%) for each emotion (*t*(29) = 12.91 for achievement, 21.57 for anger, 13.85 for fear, 19.54 for pain, 18.02 for pleasure, 13.54 for surprise, Bonferroni-corrected *p*s < 0.001, *d*s > 2.36). Of the expressions with incongruent emotion classification, positive expressions were more likely to be misclassified as negative (*t*(29) = − 5.36, *p* < 0.001, *d* = − 0.98), whereas negative expressions were equally likely to be confused within as across valences (*t*(29) = 0.95, *p* = 0.35, *d* = 0.17).

Comparing participants’ ratings on each of the six emotion scales in the *emotion rating task* (Expt2, Figs. 1, 2b), we found that the expressed emotions were rated higher on the matching scale than on the other scales (main effect of emotion scale, *F*(5, 145) = 215.55 for achievement, 178.15 for anger, 135.53 for fear, 173.63 for pain, 171.93 for pleasure and 124.06 for surprise, *p*s < 0.001; all but one pairwise comparisons contrasting the matching scale ratings with the other scale ratings were significant at *p* < 0.001; *p* = 0.04 for achievement-surprise). Above chance classification for each emotion is reported in Supplementary Table S2.

### Intensity is faithfully tracked

**Figure 3:**
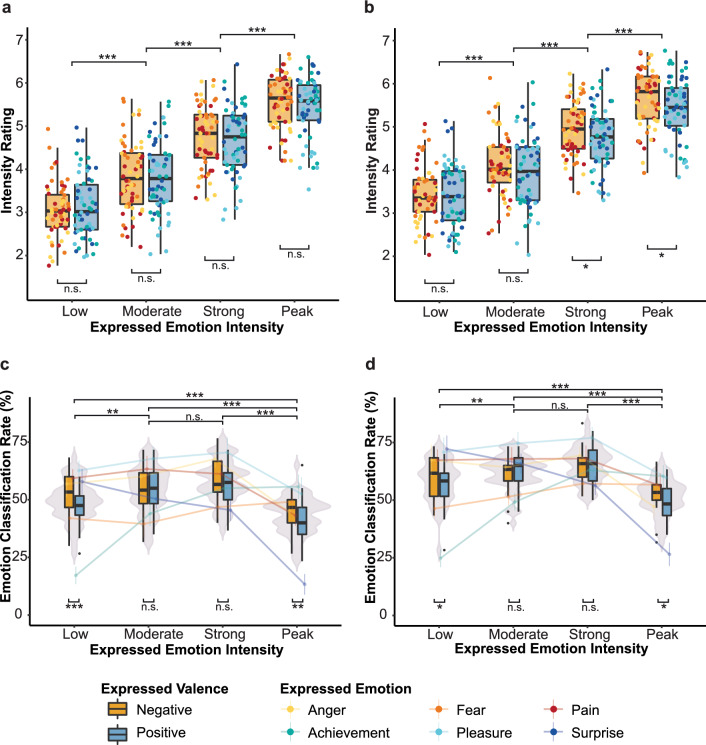
Paradoxical role of intensity. (**a**,**b**) Show positive relation of expressed and perceived intensity in Experiment 1(a) and 2 (b). Stimuli (dots) are grouped by expressed valence, emotion and intensity. (**c**,**d**) Show emotion classification accuracy as a function of valence, emotion, and intensity in Experiment 1(c) and 2(d). Violin plots represent the effect of intensity on correct emotion classification, box plots represent the interaction of valence and intensity, and lines represent the interaction of emotion and intensity on correct emotion classification. Error bars indicate 95% confidence intervals. n.s. = non-significant. *p < 0.05. **p < 0.01. ***p < 0.001. Figure created with R version 4.0.3^35^.

Congruence between expressed and perceived intensity is reflected in the monotonic increases depicted in Fig. 3a (Expt1) and Fig. 3b (Expt2). We tested whether listeners could reliably identify the intensity of the expressions and if they could do so across tasks. Separate ANOVAs were performed to investigate how listeners’ ratings vary as a function of expressed valence, emotion, and intensity.

For the Experiment 1, the Emotion × Intensity rmANOVA revealed significant main effects of emotion (*F*(5, 145) = 15.10, *p* < 0.001, *η*_p_^2^ = 0.08) and intensity (*F*(3, 87) = 266.07, *p* < 0.001, *η*_p_^2^ = 0.68), and a significant interaction (*F*(15, 435) = 9.91, *p* < 0.001, *η*_p_^2^ = 0.03). Planned comparisons confirmed systematic differences in participants’ ratings, with low (*M* = 3.09, 95% CI [2.91, 3.27]) < moderate (*M* = 3.82, [3.64, 4.00]) < strong (*M* = 4.72, [4.54, 4.90]) < peak emotion intensity ratings (*M* = 5.49, [5.31, 5.67], all *p*s < 0.001). Post hoc comparisons of the interaction are reported in Supplementary Fig. S1c.

Results were replicated in the *emotion rating task* (Fig. 3b): We found significant main effects for emotion (*F*(5, 145) = 17.05, *p* < 0.001, *η*_p_^2^ = 0.05), intensity (*F*(3, 87) = 204.00, *p* < 0.001, *η*_p_^2^ = 0.57), and a significant interaction (*F*(15, 435) = 10.85, *p* < 0.001, *η*_p_^2^ = 0.04). In line with the results from Experiment 1, planned comparisons confirmed an increase in participants’ intensity ratings from low to peak emotion intensity (*M*s = 3.41, [3.19, 3.63] < 4.03, [3.81, 4.25] < 4.85, [4.63, 5.07] < 5.54, [5.32, 5.76], *p*s < 0.001).

The effect of valence on intensity ratings was assessed in Valence × Intensity rmANOVAs. Here, results differed between the two experimental groups. In Experiment 1, intensity ratings did not differ significantly between negative (*M* = 4.30, [4.12, 4.48]) and positive expressions (*M* = 4.26, [4.08, 4.44]) (*F*(1, 29) = 0.42, *p* = 0.52). In Experiment 2, intensity ratings were higher for negative (*M* = 4.53, [4.31, 4.75]) compared to positive expressions (*M* = 4.38, [4.16, 4.60]) (*F*(1, 29) = 16.43, *p* < 0.001, *η*_p_^2^ = 0.01). As expected, the main effect of intensity was significant for both groups (Expt1, *p* < 0.001, *η*_p_^2^ = 0.71; Expt2, *p* < 0.001, *η*_p_^2^ = 0.60; *F*-ratios for intensity are reported in the Emotion × Intensity ANOVAs). The interaction of valence and intensity was significant in both groups (Expt1, *F*(3, 87) = 4.48, *p* = 0.008, *η*_p_^2^ = 0.001; Expt2, *p* = 0.007, *η*_p_^2^ = 0.002). Post-hoc comparisons revealed that for Experiment 2, differences between positive and negative valences were significant at higher intensities (*M*s = 4.75, [4.50, 5.0] and 4.94, [4.70, 5.19] (*p* = 0.04) for positive and negative strong intensity; *M*s = 5.43, [5.18, 5.68] and 5.64, [5.40, 5.89] (*p* = 0.02) for positive and negative peak intensity), but not at weaker intensities (low, *p* = 0.54; moderate, *p* = 0.11). For Experiment 1, the same trend was shown but did not reach significance.

The detected effect of expressed on perceived intensity persisted for trials in which emotion was not classified concordantly. Despite differences between congruently and incongruently classified trials (Expt1, *F*(1, 29) = 16.33, *p* < 0.001; *η*_p_^2^ = 0.02), perceived intensity increased significantly in line with intended intensity (*M*s = 3.04, 3.65, 4.68, and 5.44, with low < moderate < strong < peak, *p*s < 0.001) in trials of incongruent emotion classification, and did so also in the case of incongruent valence classification (*p* < 0.001 for all pairwise comparisons). Cumulatively, the data on intensity ratings show the coherence between expressed and perceived intensity across all tested contrasts.

### Paradoxical role of intensity reveals classification sweet spot

Separate ANOVAs were computed to assess the effect of valence, emotion, and emotion intensity on classification accuracy in Experiment 1 (Fig. 3c). Classification accuracy differed between intended emotions, *F*(5, 145) = 20.41, *p* < 0.001, *η*_p_^2^ = 0.26 and intensity levels, *F*(3, 87) = 81.66, *p* < 0.001, *η*_p_^2^ = 0.13 The interaction Emotion × Intensity was significant, *F*(15, 435) = 39.70, *p* < 0.001, *η*_p_^2^ = 0.31. Four (anger, pleasure, pain, surprise) out of six emotions featured *lower* classification accuracy for peak compared to strong, moderate, and low intensity (anger, peak < low, *p* = 0.004, pleasure, peak < low, *p* = 0.02; *p* < 0.001 for all other peak < low, moderate, strong). The opposite pattern was shown for achievement (*p*s < 0.001), whereas accuracy for fear was uniform across intensity levels.

In a Valence × Intensity rmANOVA, no main effect of valence on classification accuracy was found, *F*(1, 29) = 2.95, *p* = 0.10. Again, the main effect of intensity was significant, *p* < 0.001, *η*_p_^2^ = 0.28. Planned comparisons confirmed the pattern shown in Fig. 3c: Accuracy was highest for strong intensity expressions, *M* = 57.78%, 95% CI [55.17, 60.45], which were not significantly different from moderate intensity expressions, *M* = 54.17%, [51.5, 56.84] (*p* = 0.053). The decrease in accuracy from moderate to low intensity (*M* = 49.31%, [46.64, 51.98]) was significant (*p* = 0.004). Classification accuracy for peak intensity was lower than for strong, moderate, and low intensity *M* = 43.11%, [40.44, 45.78], *p*s < 0.001. The interaction between valence and intensity (*F*(3,87) = 5.21, *p* = 0.002, *η*_p_^2^ = 0.02) corresponded to a significant difference in accuracy between low and moderate intensity only for positive but not negative expressions, along with a significant drop in accuracy for peak compared to all other intensity levels for expressions of either valence (Fig. 3c).

In parallel to Experiment 1, separate ANOVAs were computed to assess the effect of valence, emotion, and emotion intensity on classification accuracy (Fig. 3d). The Emotion × Intensity rmANOVA revealed significant differences between emotions, *F*(5, 145) = 21.29, *p* < 0.001, *η*_p_^2^ = 0.23, intensities, *F*(3, 87) = 75.28, *p* < 0.001, *η*_p_^2^ = 0.13, and an interaction, *F*(15, 435) = 32.83, *p* < 0.001, *η*_p_^2^ = 0.33. Post hoc comparisons of the interaction replicated the pattern obtained in Experiment 1.

For the Valence × Intensity rmANOVA, no main effect of valence on classification accuracy was found, *F*(1, 29) = 1.59, *p* = 0.22. The main effect of intensity was significant, *p* < 0.001, *η*_p_^2^ = 0.32, as well as the interaction of valence and intensity, *F*(3, 87) = 3.45, *p* = 0.044, *η*_p_^2^ = 0.03. Accuracy was lower for low (*M* = 58.14%, 95% CI [55.81, 60.47]) compared to moderate intensity (*M* = 62.22%, [59.89, 64.55], *p* = 0.007), and lower for peak (*M* = 51.08%, [48.75, 53.41] compared to strong intensity (*M* = 65.14%, [62.81, 67.47], *p* < 0.001), whereas no difference was found between moderate and strong intensity (*p* = 0.095). The significant interaction stems from higher derived accuracy for expressions of negative valence at the outer intensity levels (low: positive, *M* = 55.94%, [52.99, 58.90], negative, *M* = 60.33%, [57.38, 63.29], *p* = 0.014; peak: positive, *M* = 48.83%, 95% CI [45.88, 51.79], negative, *M* = 53.33%, [50.38, 56.29], *p* = 0.012), but no significant differences at the centered intensity levels.

A second comparison across the two tasks examined how well listeners could distinguish positive from negative expressions. Valence classification accuracy (derived from forced choice judgements in Experiment 1), like emotion categorization, followed a paradoxical pattern. Accuracy was lower at peak (*M* = 68.47%) compared to strong (*M* = 76.42%, *p* < 0.001) and moderate intensity (*M* = 73.78%*, p* = 0.02), yet peak and low (*M* = 72.61%) did not differ significantly (*p* = 0.11). The highest valence confusion occurred at peak intensity expressions of positive valence, where the classification accuracy of 53.67% was only marginally above chance (50%), *t*(29) = 1.86, *p* = 0.04, *d* = 0.34. In Experiment 2, correct valence classification dropped significantly for peak (*M* = 75.56%) compared to low (*M* = 82.19, *p* = 0.001), moderate (*M* = 82.42, *p* < 0.001%, *p* = 0.004), and strong intensity (*M* = 82.67, *p* < 0.001). Again, congruency of expressed and perceived valence was lowest for positive peak emotional states (63.11%).

### Valence and arousal ratings differ

**Figure 4:**
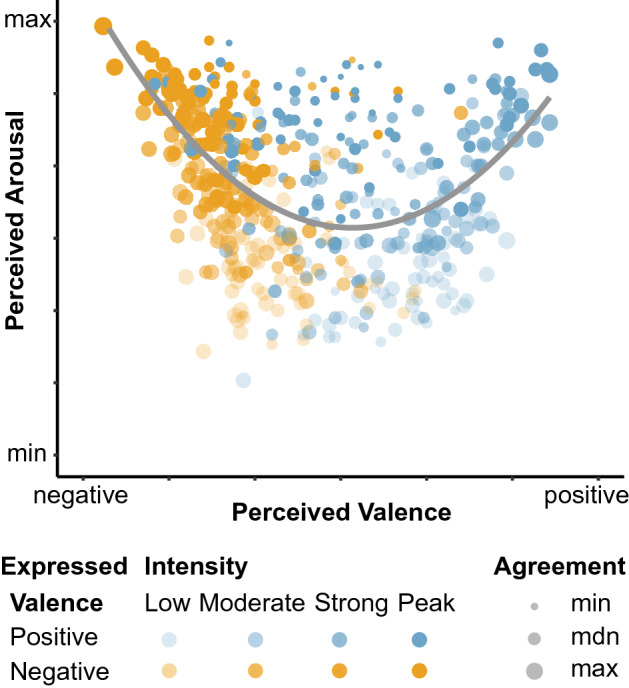
Two-dimensional space of perceived valence and arousal. Stimuli are represented by individual dots of different color (expressed valence) and transparency (expressed intensity). The boomerang-shaped distribution of valence and arousal ratings (Expt. 3) is described by their significant quadratic relationship (gray line). Dot size indicates participant agreement on valence ratings. Figure created with R version 4.0.3^35^.

Figure 4 depicts the two-dimensional space of mean valence and arousal ratings for each stimulus in the *dimensional rating task* (Fig. 1). The U-shaped distribution of affective valence and arousal can be described by a significant quadratic fit, y = 0.34*x*^*2*^ − 2.83*x* + 10, *R*^*2*^_*adj*_ = 0.23, *F*(2, 477) = 72.60, *p* < 0.001. This relationship is characterized by higher ratings in arousal for sounds which are rated as either highly pleasant or highly unpleasant. In addition, the relationship in our sample is asymmetrical: Negatively rated stimuli show higher arousal ratings (*M* = 4.82) than positively rated stimuli (*M* = 4.56), confirmed by a significant Wilcoxon test (*z* = − 2.69, *p* = 0.007).

While arousal ratings increased from low to peak intensity (*M*s = 3.63 95%, CI [3.41, 3.85] > 4.33, [4.11, 4.55]) > 5.13, [4.92, 5.35] > 5.79, [5.57, 6.01], *p*s < 0.001), the pattern of valence ratings showed interesting confusion and variation in participants’ agreement (Fig. 4). The number of expressions perceived as negative (299, average rating < 4) and positive (181, average rating ≥ 4) deviated significantly from the balanced number of stimuli per expressed valence (*X*^*2*^ (1, *N* = 480) = 183.91, *p* < 0.001). A factorial logistic regression quantified the effect of expressed valence and intensity on congruent or incongruent valence rating. Positive expressions, especially high and peak intensity expressions, were more likely to be rated of negative valence (strong, *z* = − 2.08, *p* = 0.04; peak, *z* = − 3.13, *p* = 0.002), accounting for the higher number of stimuli perceived as negative.

## Discussion

Three experiments show that listeners are remarkably good at inferring meaning from variably intense nonverbal vocalizations. Yet their ability to do so is affected by the expressed emotional intensity. We demonstrate a complex relationship between intensity and inferred affective state. Whereas both intensity and arousal are perceived coherently over the range of expressed intensities, the facilitatory effect of increasing intensity on classifying valence and emotion category plateaus at strong emotions. Remarkably, peak emotions are the most ambiguous of all. We call this the ‘emotion intensity paradox’. Our results suggest that value (i.e., valence and emotion category) cannot be retrieved easily from peak emotion expressions. However, arousal and emotion intensity *are* clearly perceivable in peak expressions.

In addition to the reported parabolic relationship of emotional intensity to classification accuracy, overall accuracy scores of individual emotions, although above chance, were far from perfect and in fact relatively low compared to previous research^1,3,34^. A direct comparison of accuracy scores across studies should be treated with caution, as, for example, the number of emotion categories varies across studies, and so does their intensity—here shown systematically to affect emotion classification. Furthermore, substantial differences exist in the tested stimulus sets themselves, that is stimulus production and selection procedures as well as stimulus sources (i.e., studio-produced or real-life). One speculative, but interesting possibility is that the lower convergence observed in our data reflects the heterogeneity allowed for in the stimulus material.

The data are incompatible with the view of diagnostic emotion expression suggested by basic emotion theories^36,37^. Likewise, the data challenge the conception that valence and arousal are equivalent elements in the composition of core affect^15,16^. Future work will need to investigate whether valence and arousal really share the same level of representation. Information on arousal is already available at early processing stages^38–41^ and may serve as an attention-grabbing filter, ensuring the detection of biological relevance in the most extreme cases. Valuation likely constitutes a more complex process, perhaps secondary in peak emotion.

We exploited a new database of human vocal emotion expressions (http://doi.org/10.5281/zenodo.4066235), systematically manipulating emotion intensity. In line with previous research^3,4^, the data underscore that emotion intensity constitutes a prominent property of vocal emotion communication. In our population of listeners (the cultural relativity of vocal emotion perception is discussed e.g., in Ref.^18^), we report compelling specific effects of intensity. Forced choice judgements and emotion ratings both revealed an inverted U pattern: The expressed emotion was most accurately classified for moderate and strong intensity expressions; low intensity expressions were frequently confused, and the *least* accurately classified were peak intensity expressions. The higher ambiguity of peak states was reflected in both lower valence and lower emotion classification accuracy. At the most extreme instances of emotion, the evaluation of ‘affective semantics’, i.e., valence and emotion type, is constrained by an ambiguous perceptual representation.

We find that peak emotion is not per se ambiguous. Arousal and intensity of emotion expressions are perceived clearly across the range of expressed intensities, including peak emotion (e.g. Fig. 4). Notably, we find that the intensity of the expressions is accurately perceived even if other affective features, such as valence and emotion category, prove ambiguous.

In other words, for a given expression, despite the unreliable identification of the *affective semantics*, the *relevance* of the signal is readily perceived, through the unambiguous representation of arousal and intensity. Taken together, extremely intense expressions seem to convey less information on the polarity (positive or negative, triumph or anger), though their indication of ‘relevance’ remains unaltered. We speculate that this central representation of ‘alarmingness’, i.e., biological relevance of highly intense expressions, comes at the cost of other *affective semantics,* including valence and type of affective state. The latter might rely on contextual cues, underlining the role of top-down modulations and higher-order representations of emotional states^12,42,43^.

In nonverbal vocalizations, the effects of increased emotional intensity and arousal have been linked to acoustic characteristics that attract attention^25,27,44^. Screams, for example, have spectro-temporal features irrelevant for linguistic, prosodic, or speaker identity information, but dedicated to alarm signals. The corresponding unpleasant acoustic percept, roughness, correlates with how alarming the scream is perceived and how efficiently it is appraised^26^. One hypothesis that arises is that information is prioritized differently as a function of emotion intensity. At peak intensity, the most vital job is to detect ‘big’ events. A salient, high arousal signal may serve as an attention-grabbing filter in a first step, and affective semantic evaluation may follow. In contrast, intermediate intensity signals do not necessarily elicit or require immediate action and can afford a more fine-grained analysis of affective meaning. A possible neurobiological implementation is that information is carried at different timescales, and ultimately integrated in a neural network underlying affective sound processing^39,41,45^. Concurrent functional pathways allow a rapid evaluation of relevance for vocal emotions of *any* valence, occurring at early processing stages and via fast processing routes^38–41,46,47^. Though perceptually unavailable, it might well be that information is objectively present in the signal, as has been shown for facial and body cues of extreme emotion^11^. The conjecture that a similar pattern might also exist in vocal peak emotion would resonate with the interpretation of the findings as temporally masked affective value and emotion information through the central representation of salience—via arousal and emotion intensity.

## Materials and methods

### Study design

#### Stimuli

The stimuli are 480 nonverbal vocalizations, representing the Core Set of a validated corpus^48^. The database comprises six affective states (three positive and three negative) at four different intensity levels (low, moderate, strong, and peak emotion intensity; note that in this text, the term “intensity” exclusively refers to the *emotional* intensity, i.e., the variation from a very mildly sensed affective state to an extremely intense affective state and should not be confused with the auditory perception of signal intensity as *loudness*). The six affective states—achievement/triumph, anger, fear, pain, positive surprise, sexual pleasure-represent a suitable, well-studied sample of affective states for which variations in emotion intensity have previously been described^3,4,10^.

Vocalizations were recorded at the Berklee College of Music (Boston, MA). Ten female speakers, all non-professional actors, were instructed to produce emotion expressions as spontaneously and genuinely as possible. No restrictions were imposed on the specific sounds speakers should produce, only that vocalizations should have no verbal content as in words (e.g., “yes”) or interjections (e.g., “ouch”). Following a technical validation, the Core Set was developed as fully crossed stimulus sample based on authenticity ratings. Stimuli were recorded with a sampling rate of 44.1-kHz (16-bit resolution). Sound duration ranges from 400 to 2000 ms.

#### Participants

A total of ninety participants were recruited through the Max-Planck-Institute for Empirical Aesthetics (MPIEA), Frankfurt. Thirty participants were assigned to the *emotion categorization task* (*M* = 28.77 years old, *SD* = 9.46; 16 self-identified as women, 14 as men); thirty (*M* = 28.53 years old, *SD* = 8.62; 15 self-identified as women, 14 as men, 1 as nonbinary) to the *emotion rating task*; and thirty participants (*M* = 24.37 years old, *SD* = 4.80; 15 self-identified as women, 15 as men) to the *dimensional rating task* (Fig. 1). Our sample size was based on previous research^3,23^, and a power analysis in G*Power^49^ confirmed that our sample size (*N* = 30 each) would allow us to detect an effect as small as *η*_p_^2^ = 0.005 (Cohen’s *f* = 0.06) with a power of 0.80. The experimental procedures were approved by the Ethics Council of the Max Planck Society. Experiments were performed in accordance with relevant named guidelines and regulations. Participants provided informed consent before participating and received financial compensation. All participants were native speakers of German, reported normal hearing, and no history of psychiatric or neurological illnesses.

#### Procedure

The studies took place at MPIEA. The 480 stimuli were presented using Presentation (Version 20.0) software (www.neurobs.com), through DT 770 Pro Beyerdynamic headphones. Sound amplitude was calibrated to a maximum of 90.50 dB(A), resulting in 43 dB(A) for the peak amplitude in the quietest sound file. Each stimulus was presented once in pseudorandomized order. No feedback regarding response accuracy was provided.

#### Emotion categorization task (Experiment 1)

Participants were asked to assign one of seven possible response options to each vocalization: the German emotion labels for anger (Ärger), fear (Angst), pain (Schmerz), achievement (Triumph), positive surprise (Positive Überraschung), and sexual pleasure (Sexuelle Lust), plus a ‘none of the specified emotions’ option (Keines der Genannten). Next, participants were asked to indicate how intensively they believed the speaker had experienced the emotional state, reaching from minimally intense (“minimal”) to maximally intense (“maximal”). Finally, participants indicated how authentic they perceived the expression, from not at all (“gar nicht”) authentic to fully (“vollkommen”) authentic. The order of the 7AFC and intensity rating tasks was counterbalanced across participants. After the authenticity rating, the next stimulus was played automatically.

#### Emotion rating task (Experiment 2)

Participants completed ratings for each emotion. They were instructed to indicate how clearly they perceived the specified emotion in the expression. A judgement from not at all (“gar nicht”) to completely (“völlig”) was performed on each of the simultaneously presented scales. Thereby, from none to all emotions could be identified to various extents in *each* vocalization. As in the categorization task, emotion intensity was rated. The order of emotion ratings and emotion intensity ratings was counterbalanced across participants.

#### Dimensional rating task (Experiment 3)

Participants were asked to judge each stimulus on the dimensions valence and arousal. Valence was rated from negative to positive and arousal from minimal to maximal. The scales were presented subsequently on individual screens, in counterbalanced order across participants. Authenticity judgements were performed in the same format as described for the categorization task.

#### Statistical analysis

All statistical analyses and data visualizations were performed using R Studio.

We refer to “classification accuracy” as the consistency of speaker intention and listener perceptual judgements. In Experiment 1, this corresponds to the percentage of correct classification of emotions. A measure of accuracy was also obtained from the emotion ratings performed in Experiment 2 by defining the response as a match whenever the highest of the ratings was provided on the intended emotion scale, and a miss whenever rated lower on the intended than on any other scale. As additional indices that take into account response biases, we report unbiased hit rates, differential accuracy, false alarm rates, and detailed confusion matrices of the response data in the Supplemental Information.

Intensity ratings and classification accuracy were tested with repeated measures analyses of variance (rmANOVA) to assess the effects of affective stimulus properties (i.e., valence, emotion category, and emotion intensity) and their interactions. Normality was screened; sphericity was assessed using Mauchly’s sphericity tests. When sphericity could not be assumed (*p* < 0.001), Greenhouse–Geisser corrections were applied. For readability, we report uncorrected degrees of freedom and adjusted *p* values. Pairwise comparisons were adjusted using the Tukey’s *HSD* correction in the *emmeans* package^50^.

Authenticity ratings are reported and discussed in the Supplemental Materials. Perceptual judgements for each stimulus are available at https://osf.io/jmh5t/.

## Supplementary Information

Supplementary Information.

## Data Availability

The data is available via the Open Science Framework at https://osf.io/jmh5t/. All stimuli are openly available at http://doi.org/10.5281/zenodo.4066235.
