# Supplementary material for: The paradoxical role of emotional intensity in the perception of vocal affect
Source: Sci Rep. 2021 May 6;11:9663. doi: 10.1038/s41598-021-88431-0 (PMC8102532; doi:10.1038/s41598-021-88431-0)
Supplement: Supplementary file 1 — Supplementary Information. [file 41598_2021_88431_MOESM1_ESM.docx]

The paradoxical role of emotional intensity in the perception of vocal affect

N. Holz^1*^, P. Larrouy-Maestri^1,2^, D. Poeppel^1,2,3^

^1^ Department of Neuroscience, Max Planck Institute for Empirical Aesthetics, Frankfurt/M., Germany

^2^ Max Planck NYU Center for Language, Music, and Emotion, Frankfurt/M., Germany, New York, USA

^3^ Department of Psychology, New York University, New York, NY, USA

* Corresponding author

Email: natalie.holz@ae.mpg.de

**Supplementary Materials**

**Supplemental methods**

**Interrater reliability.** Interrater Reliability for categorical ratings was estimated using Light Kappa [51], a common kappa-like variant, which is suitable for studies with fully crossed designs [52]. Kappa scores, computed as the arithmetic mean of all kappa between rater pairs, reflect the degree of agreement between raters in emotion classification. Reliability of ratings was measured with intraclass correlations separately for each scale (intensity, arousal, valence, and authenticity). Intra-class correlation coefficients (ICCs) were computed using a two-way random effects model and a consistency definition using average-measures [52]. Reliability estimates were computed using the irr package [53] and are reported in Table S1.

**Additional accuracy indices.** In addition to raw classification accuracy scores, we report differential accuracy and false alarm rates. These measures, taking into account response biases, are then reflected in the unbiased hit rates and corrected chance values. The unbiased hit rate (HU [54]) is defined as the joint probability of the uncorrected hit rate and the differential accuracy, namely, that a stimulus category is correctly identified (given the frequency of it being presented), and that a response category is correctly used (given the frequency of it being used). It is thus calculated as the squared frequency of correct identifications of a given emotion (nominator), divided by the number of stimuli in the same category, multiplied by the total number of times that the corresponding category was used across all categories (denominator). The HU score varies between 0, no correct response, and 1, perfect performance, and except for the case of perfectly unbiased accuracy, has a smaller value than the uncorrected accuracy index. Furthermore, detailed confusion matrices of the response data are provided (Figure S1), as these are proposed as the most certain way to avoid bias [1, 23].

**Supplemental results**

**Emotion classification.** Correct classification and confusion patterns are reported in Table S2 (Experiment 1) and Table S3 (Experiment 2).

To compare ratings between the forced choice task and the emotion ratings, we report response patterns visualized in confusion matrices for each task (Figure S1a and b). The matrices display the cumulative frequency scores and rating scores, respectively, for each combination of expressed and perceived emotion at each intensity (low to peak and 1–7) across all trials. By visual inspection, similar patterns of concordant and discordant classification can be identified across tasks. These descriptive results are supported by a high positive correlation (Pearson’s *r* = .89, *p* < .001) between forced choice responses and emotion ratings across all possible pairs of expressed and perceived emotion.

**Authenticity ratings.** Participants’ authenticity ratings indicated that expressions were perceived as rather authentic, as revealed by separate one-sample t-tests with *M* = 4.51 (95% CI [4.31, 4.72], *t*(29) = 5.03; *p* < .001) for Experiment 1, and *M* = 4.59 ([4.39, 4.80], *t*(29) = 5.71; *p* < .001) for Experiment 3. An independent t-test suggests that the average ratings of the two groups did not significantly differ (*t*(58) = 0.53, *p* = .60). Agreement among raters was high within and across the two groups (ICC (C, 60) = .90, [.88, .91], (ICC (C, 30) = .82, [.80, .84], for Experiment 1 and 3). We thus evaluated authenticity on the combined ratings of both experiments (*N* = 60). The average authenticity ratings per stimulus ranged from 2.08 to 5.98 following a left-skewed distribution (*M* = 4.55, *SD* = 0.60, *Mdn* = 4.63).

Next, we tested if stimuli in all conditions (i.e., positive as well as negative expressions, of any of the emotion categories, at any intensity level) were perceived as authentic. In every condition, stimuli obtained higher than neutral average authenticity ratings, indicating their perception as rather authentic (*p* < .001 for all but one (achievement—*p* = .05) comparisons in one-sided Wilcoxon signed-rank tests; see Table S4).

Additionally, we tested for differences in authenticity ratings between correct and incongruent emotion classification separate for the forced choice raters of Experiment 1. In a Wilcoxon-Mann-Whitney test we found significantly higher ratings for correctly classified (*M* = 4.72) than for misclassified sounds (*M* = 4.30, *z* = –15.39, *p* < .001). However, Wilcoxon signed-rank tests confirmed that stimuli, correctly classified or not, were perceived as rather authentic (correct classification: *z* = 32.88, *p* < .001; incorrect classification: *z* = 13.71, *p* < .001).

**
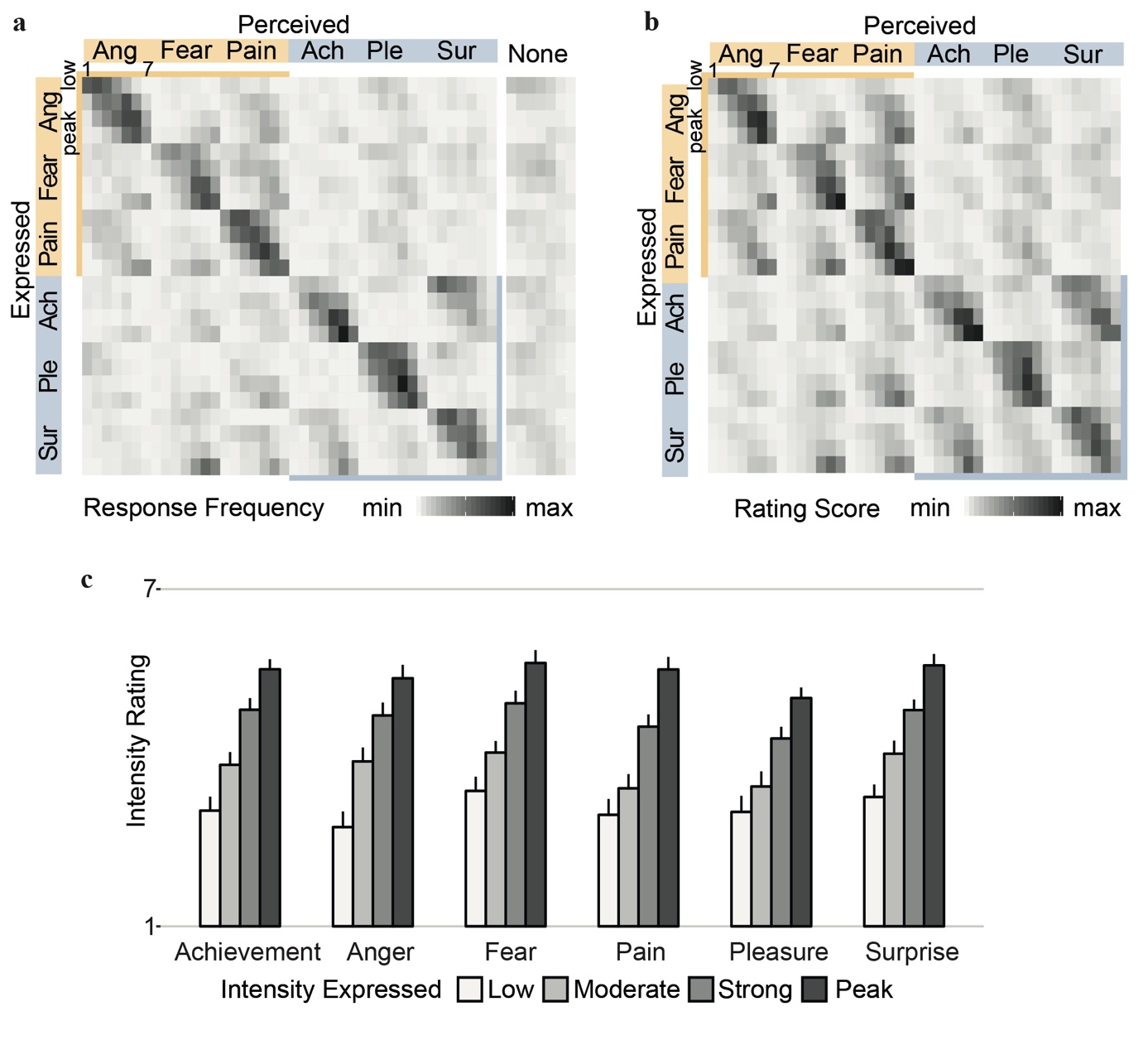
**

**Fig S1. Emotion classification and intensity ratings.**

Top panels: classification and confusion patterns for Experiment 1**(a)** and 2 **(b)**. The main diagonal shows correct classification for main emotion categories (squares) and Emotion x Intensity combinations (tiles). The top left quadrant contains confusions within negative valence, the bottom right quadrant confusions within positive valence, respectively. Bottom panel **(c)**: intensity ratings as a function of expressed intensity for each emotion in Experiment 1. Error bars represent 95% confidence intervals. For each of the six emotions, ratings were monotonically peak > strong > moderate > low intensity expressions, with a smaller effect in the low to moderate condition for pain (*p* = .02) and pleasure (*p* = .03) compared to all other comparisons (*p* < .001). Figure created with R version 4.0.3 [35].

Ach = Achievement; Ang = Anger; Ple = Pleasure; Sur = Surprise.

| Rating scale | ICC  average (C, 30) | 95% CI | ICC  single (C, 1) | 95% CI | *F* ratio | *p* |
| --- | --- | --- | --- | --- | --- | --- |
| Intensity (Exp. 1) | .97 | [.97, .97] | .51 | [.48, .55] | 32.30 ^a^ | < .001 |
| Intensity (Exp. 2) | .97 | [.96, .97] | .49 | [.46, .52] | 29.50 ^a^ | < .001 |
| Intensity (Exp. 1+2) | .98 | [.98, .99] | .50 | [.47, .53] | 60.60 ^b^ | < .001 |
| Valence (Exp. 3) | .97 | [.96, .97] | .50 | [.47, .53] | 30.80 ^a^ | < .001 |
| Arousal (Exp. 3) | .96 | [.96, .97] | .46 | [.43, .50] | 26.90 ^a^ | < .001 |
| Achievement (Exp. 2) | .98 | [.98, .98] | .61 | [.58, .64] | 48.30 ^a^ | < .001 |
| Anger (Exp. 2) | .97 | [.97, .98] | .54 | [.51, .58] | 36.50 ^a^ | < .001 |
| Fear (Exp.2) | .97 | [.97, .97] | .51 | [.48, .54] | 32.40 ^a^ | < .001 |
| Pain (Exp.2) | .97 | [.96, .97] | .49 | [.46, .52] | 29.60 ^a^ | < .001 |
| Pleasure (Exp.2) | .97 | [.96, .97] | .51 | [.47, .54] | 31.70 ^a^ | < .001 |
| Surprise (Exp.2) | .97 | [.97, .97] | .52 | [.48, .55] | 32.90 ^a^ | < .001 |
| Authenticity (Exp. 1) | .82 | [.80, .84] | .13 | [.12, .15] | 5.58 ^a^ | < .001 |
| Authenticity (Exp. 3) | .82 | [.80, .84] | .13 | [.12, .15] | 5.58 ^a^ | < .001 |
| Authenticity (Exp. 1+3) | .90 | [.88, .91] | .12 | [.11, .14] | 9.49 ^b^ | < .001 |

**Table S1. Interrater reliability of rating scales in Experiment 1, 2, and 3.**

*Note.* Intra-class correlation coefficients (ICCs) were computed under a consistency definition using average-measures (column 2 and 3) and single-measures (column 4 and 5). Significance was indicated by a two-way random effects model. Fleiss Kappa for emotion classification was *k* = .47, indicating moderate interrater agreement.

^a^ *F*(479, 13891). ^b^ *F*(479, 28261).

|  | | Perceived emotion chosen by individual raters, % | | | | | | | | | | | | False Alarms | | H_u_, % | | Corr. Chance, % | |
| --- | --- | --- | --- | --- | --- | --- | --- | --- | --- | --- | --- | --- | --- | --- | --- | --- | --- | --- | --- |
|  | | Ang | | | Fear | Pain | | Ach | Ple | | Sur | | None |  |  | |  | |  |
| Expressed emotion | | |  |  | | |  |  | |  | |  |  |  | |  | |  | |
|  | Ang | | **57.33***** | 6.08 | | | 14.50 | 3.04 | | 8.08 | | 2.75 | 8.21 | 8.13 | | 33.54 | | 2.72 | |
|  | Fear | | 9.75 | **44.38***** | | | 17.62 | 2.92 | | 9.04 | | 5.83 | 10.46 | 8.25 | | 23.00 | | 2.38 | |
|  | Pain | | 16.21 | 7.92 | | | **56.75***** | 2.08 | | 8.12 | | 2.75 | 6.17 | 11.98 | | 27.60 | | 3.24 | |
|  | Ach | | 4.29 | 7.04 | | | 6.50 | **42.96***** | | 2.50 | | 28.67*** | 8.04 | 4.94 | | 27.27 | | 1.88 | |
|  | Ple | | 7.04 | 4.71 | | | 11.17 | 1.04 | | **63.38***** | | 5.54 | 7.12 | 6.53 | | 41.82 | | 2.67 | |
|  | Sur | | 3.38 | 15.50 | | | 10.12 | 15.62 | | 4.92 | | **41.75***** | 8.71 | 9.11 | | 19.97 | | 2.42 | |
| Total | | |  |  | | |  |  | |  | |  |  |  | | 28.86 | | 2.55 | |

**Table S2.** **Correct classification and confusion patterns (Expt1).**

*Note.* The main diagonal in bold represents correct classification. ****p* < .001 for hit and confusion rates higher than expected by chance (16.67%). Unbiased hit rates (H_u_) were tested against corrected chance values (*t*(29) = 16.91 for achievement, 25.00 for anger, 18.74 for fear, 20.08 for pain, 27.43 for pleasure, 18.02 for surprise; all significant after Bonferroni correction, *p* < .001). False alarm rates were the highest for pain, explaining the comparably large decrease from uncorrected accuracy rate to the H_U_. Contrarily, response bias (i.e., comparably low false alarm rate along with high differential accuracy) for achievement was relatively low, accounted for by the smaller difference of raw and unbiased hit rate.

H_u_ = unbiased hit rate, all above chance. Ach = Achievement; Ang = Anger; Ple = Pleasure; Sur = Surprise.

|  |  | Perceived emotion index, % | | | | | | | | | | | Mean derived accuracy, % | | *SD*, derived accuracy | |
| --- | --- | --- | --- | --- | --- | --- | --- | --- | --- | --- | --- | --- | --- | --- | --- | --- |
|  |  | Ang | Fear | | Pain | | Ach | | Ple | | Sur | |  |  |  |  |
| Expressed | |  | |  | |  | |  | |  | |  |  |  | |  |
|  | Ang | **58.63***** | | 23.29 | | 35.92 | | 19.36 | | 25.39 | | 19.50 | 62.04 | 0.09 | |  |
|  | Fear | 26.54 | | **54.32***** | | 41.09 | | 18.46 | | 25.77 | | 22.70 | 52.92 | 0.10 | |  |
|  | Pain | 34.36 | | 28.67 | | **62.05***** | | 17.62 | | 27.24 | | 18.62 | 64.83 | 0.12 | |  |
|  | Ach | 20.15 | | 22.11 | | 22.93 | | **55.54***/*** | | 20.43 | | **51.30*** | 49.33 | 0.08 | |  |
|  | Ple | 20.93 | | 22.82 | | 32.49 | | 18.80 | | **63.51***** | | 23.96 | 70.54 | 0.10 | |  |
|  | Sur | 20.16 | | 28.27 | | 26.94 | | 37.53 | | 24.19 | | **54.82***** | 55.21 | 0.09 | |  |

**Table S3. Correct classification and confusion patterns (Expt2).**

*Note.* The Perceived emotion index is the average rating on each 7-point scale, rescaled to percent. The main diagonal in bold displays ratings on matching scales (expressed emotion = perceived emotion). Planned comparisons for higher ratings on matching scale than on other scale were significant at *p* = .04 (*) for expressed achievement–perceived surprise, and *p* < .001 (***) for all other comparisons. For derived accuracy scores, hit was defined as highest rating on matching scale for each target emotion, and miss was defined as highest rating on other scale. Ach = Achievement; Ang = Anger; Ple = Pleasure; Sur = Surprise.

| Factor | Condition | Authenticity rating, *M* | Comparison to neutral authenticity | |
| --- | --- | --- | --- | --- |
|  |  |  | *z* score | *p* value |
| Valence ^a^ | Positive | 4.40 | 8.66 | < .001 |
|  | Negative | 4.71 | 12.62 | < .001 |
| Emotion ^b^ | Achievement | 4.11 | 1.67 | .048 |
|  | Pleasure | 4.73 | 7.75 | < .001 |
|  | Surprise | 4.37 | 5.14 | < .001 |
|  | Anger | 4.73 | 7.48 | < .001 |
|  | Fear | 4.63 | 6.78 | < .001 |
|  | Pain | 4.77 | 7.45 | < .001 |
| Intensity ^c^ | Low | 4.73 | 8.69 | < .001 |
|  | Moderate | 4.42 | 6.07 | < .001 |
|  | Strong | 4.47 | 7.19 | < .001 |
|  | Peak | 4.59 | 8.22 | < .001 |

**Table S4. Authenticity ratings (Expt1 and Expt3).**

*Note.* Statistical comparisons of average authenticity ratings in each condition to neutral authenticity (corresponding to the center of the 7-point Likert-scale, 4) in one-sided Wilcoxon signed-rank tests. Across all levels of valence, emotion and intensity, expressions were perceived as authentic.

^a^ *df* = 239. ^b^ *df* = 79. ^c^ *df* =119

**Supplemental discussion**

Authenticity judgements were collected alongside affective evaluations, as previous work suggests effects of emotional intensity on how authentic expressions are perceived [55–58]. Furthermore, the current study relies on a set of acted stimuli, and studio-produced expressions may differ from real-life expressions [55, 57, 59]; it is thus important to get a clear sense of the perceived authenticity of the materials. Notably, the naturalness or artificiality of a stimulus is not simply a consequence of being a real-life versus a studio recorded vocalization. Natural settings do not per se entail genuineness and authenticity, as they are to some degree shaped by strategic aims as well as sociocultural control and regulation [1, 59, 60]. Actor portrayals, just like naturally occurring expressions, may contain both spontaneous and posed aspects [1, 24]. Though in the given setup it is difficult to assess to what extent our stimulus material truly mirrors genuine emotion expressions, authenticity ratings provide relevant information for the interpretation of paradoxical emotion perception. Our results show that low and peak intensity expressions—the least accurately classified—were rated as more authentic than moderate and strong intensity expressions. As such, it is rather unlikely that peak intense expressions solely constitute exaggerated caricatures [56]. In lieu, our data suggest that the relation of emotional intensity and emotion classification lies beyond the perceived authenticity of the signal.

**References**

1. Light, R. J. Measures of response agreement for qualitative data: some generalizations and alternatives. *Psychol. Bull*. **76**, 365–377. <https://doi.org/10.1037/h0031643> (1971).
2. Hallgren, K. A. Computing inter-rater reliability for observational data: an overview and tutorial. *Tutor. Quant. Methods Psychol*. **8**, 23–34 (2012).
3. Gamer, M., Lemon, J., Fellows, I. & Singh, P. Irr: various coefficients of interrater reliability and agreement. R package version 0.84.1[software] (2012).
4. Wagner, H. L. On measuring performance in category judgment studies of nonverbal behavior. *J. Nonverbal Behav*. **17**, 3–28. <https://doi.org/10.1007/BF00987006> (1993).
5. Anikin, A. & Lima, C. F. Perceptual and acoustic differences between authentic and acted nonverbal emotional vocalizations. *Q. J. Exp. Psychol.* **71**, 622–641. <https://doi.org/10.1080/17470218.2016.1270976> (2018).
6. Calder, A. J. *et al.* Caricaturing facial expressions. *Cognition* **76**, 105–146. <https://doi.org/10.1016/S0010-0277(00)00074-3> (2000).
7. Juslin, P. N., Laukka, P., & Bänziger, T. The mirror to our soul? Comparisons of spontaneous and posed vocal expression of emotion. *J. Nonverbal Behav.* **42**, 1–40. <https://doi.org/10.1007/s10919-017-0268-x> (2018).
8. Lavan, N., Scott, S. K. & McGettigan, C. Laugh like you mean it: Authenticity modulates acoustic, physiological and perceptual properties of laughter. *J. Nonverbal Behav.* **40**, 133–149. <https://doi.org/10.1007/s10919-015-0222-8> (2016).
9. Bänziger, T. & Scherer, K. R. Using actor portrayals to systematically study multimodal emotion expression: the GEMEP corpus in *Affective computing and intelligent interaction* (eds. Paiva, A. C. R. *et al.*) 476–487 (Springer, 2007). <https://doi.org/10.1007/978-3-540-74889-2_42>
10. Banse, R. & Scherer, K. R. Acoustic profiles in vocal emotion expression. *J. Pers. Soc. Psychol.* **70**, 614–636. <https://doi.org/10.1037/0022-3514.70.3.614> (1996).
